# Hormones in Breast Milk and Effect on Infants’ Growth: A Systematic Review

**DOI:** 10.3390/nu11081845

**Published:** 2019-08-09

**Authors:** Alessandra Mazzocchi, Maria Lorella Giannì, Daniela Morniroli, Ludovica Leone, Paola Roggero, Carlo Agostoni, Valentina De Cosmi, Fabio Mosca

**Affiliations:** 1 Department of Clinical Sciences and Community Health, University of Milan, 20122 Milan, Italy; 2 Fondazione IRCCS Ca’ Granda Ospedale Maggiore Policlinico, NICU, 20122 Milan, Italy; 3 Fondazione IRCCS Ca’ Granda Ospedale Maggiore Policlinico, Pediatric Intermediate Care Unit, 20122 Milan, Italy

**Keywords:** hormones, adipokines, breast milk, growth, body composition, term infant

## Abstract

Breast milk is characterized by a dynamic and complex composition which includes hormones and other bioactive components that could influence infant growth, development, and optimize health. Among the several beneficial effects associated with prolonged breastfeeding, a 13% decrease in the risk of overweight and obesity has been reported. Recent research has focused on breast milk hormones contributing to the appetite and energy balance regulation and adiposity. Accordingly, we conducted a literature systematic review with the aim to provide an update on the effect of leptin, ghrelin, Insulin Growth Factor 1, adiponectin, and insulin on infants’ and children’s growth and body composition. The revised literature reveals contrasting findings concerning the potential role of all these hormones on modeling growth and fat mass apposition and health outcomes later in life. Further studies are needed to gain further insight into the specific role of these bioactive components in metabolic pathways related to body composition. This could help gain a further insight on infants’ growth, both in physiological and pathological settings.

## 1. Introduction

Breast milk (BM) is the only species-specific human food tailored to meet infants’ needs through the adaptation of its micro and macronutrient content and bioactive components, depending upon the mother–infant dyad characteristics (i.e., gestational age at birth, birth weight, etc.) [1,2].

Furthermore, the dynamic composition of BM also allows mother–infant signaling over lactation [3,4]. As a result, the infant is guided in the developmental and physiological processes through breastfeeding, whose crucial role in promoting healthy growth and optimal cognitive development is widely acknowledged [2,5]. Breastfeeding is beneficial through an epigenetic effect that is demonstrated to be dose-dependent [6]. However, although our understanding of the bioactive, non-nutritional compounds of BM and their dynamic changes over time has improved, the complexity of BM composition and the synergistic mechanisms responsible for its life-long biological effects have not yet been unraveled [7]. Among the several beneficial health effects associated with prolonged breastfeeding, a 13% decrease in the risk of overweight and obesity development has been reported [8]. In light of the well-known different growth trajectory and body composition development that characterize breastfed infants in comparison to formula-fed ones [9,10], gaining further insight into the factors that contribute to infants’ appetite regulation and metabolic programming in the long run is, therefore, crucial for developing adequate strategies aimed at obesity prevention [11,12]. Accordingly, recent research has focused on BM hormones involved in the regulation of appetite and energy balance and adiposity, with the aim of identifying the factors that modulate their concentration throughout lactation and elucidating their potential biological relevance within this context [13,14]. We conducted a literature review with the aim of providing an update on the effects of BM leptin, ghrelin, Insulin Growth Factor 1 (IGF-1), adiponectin, and insulin found within BM on infants’ and children’s growth and body composition.

## 2. Methods

We performed a review using PUBMED, searching for all trails published in English from 2009 up to July 2019. The following key words were used: “Leptin” OR “Ghrelin” OR “IGF-1” OR “Adiponectin” OR “Insulin” OR “Adipokines” AND “BM” OR “donor milk” OR “banked milk” AND “weight gain” OR “body composition” OR “obesity” OR “adiposity” OR “growth” AND “infant” OR “children”. In this review, observational studies examining the effects of these BM hormones on infants’ and children’s anthropometry and growth were selected. A total of 344 articles were initially identified and, among them, 30 studies were retrieved and evaluated for inclusion as relevant studies for the analysis by reviewing the abstract, and, when necessary, the full text. A manual bibliographic cross-referencing was also performed. We also reviewed the reference lists of relevant studies in order to detect other relevant primary sources.

**Figure 1:**
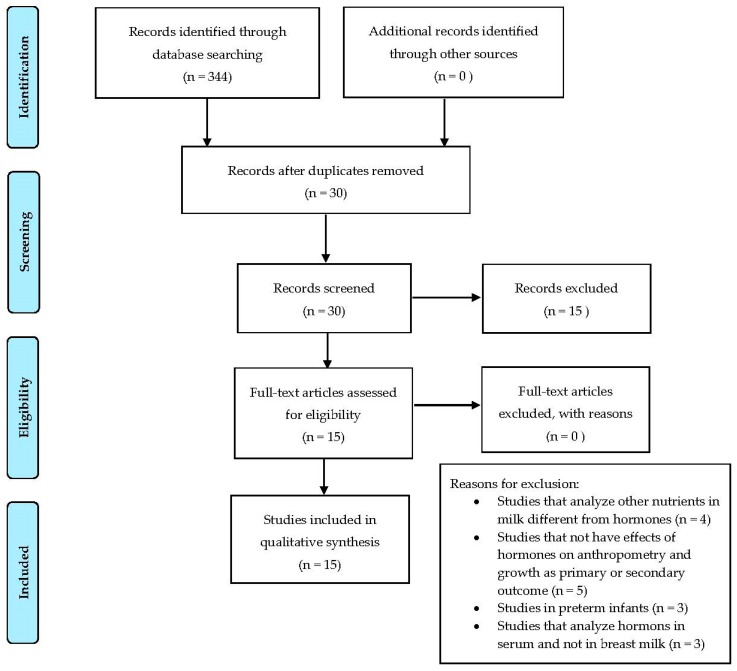
PRISMA diagram of search strategy.

Articles were considered as relevant and included in the analysis if (a) they reported the effect of milk hormones on infant growth; (b) included weight gain, body composition, obesity, adiposity as outcome measures; and (c) enrolled human participants. The selection procedure used for identifying and including the studies is summarized in Figure 1. Finally, we included 15 observational studies for the review. Three researchers independently searched, screened, and identified studies and abstracted and tabulated data. Discrepancies were addressed and sorted out by discussion.

## 3. Results

Young et al. [15] performed a prospective study of 41 healthy, term, breastfed infants, with the aim of assessing components in BM that are related with infant growth and may affect fat-free mass (FFM) or fat mass (FM) deposition thorughout the first four months of life. A significant association between the trajectory of weight for length Z-scores (WLZ) and BM insulin was detected. However, the effect differed by maternal body mass index (BMI), being the association negative among infants of normal weight mothers. In the multivariable model, mean BM insulin quintiles were significantly associated with infant fat gain rate, suggesting the role of this hormone in regulating fat-mass accretion. On the contrary, an inverse association among BM adiponectin content and rate of fat gain was found.

Kon et al. [16] assessed 103 mother–infant pairs during the first three months of lactation and reported higher BM concentration of IGF-1 in the mothers of infants with high weight gain than in the mothers of those with low and normal weight gain at all study points. BM leptin and ghrelin content tended to show a similar behavior at two and three months of lactation and at one and two months of lactation, respectively. These results suggest that both leptin and ghrelin contribute to infants’ weight gain. On the contrary, in another prospective study, Yis et al. [17] found that neither BM ghrelin nor BM leptin was correlated with anthropometric data in breastfed infants. They only observed positive correlations when they analyzed hormones sampled in infants’ serum, in particular between ghrelin and triceps skinfold thickness (TSF), and leptin and weight, TSF and weight gain at three months of age. Gridneva and colleagues [18] showed a different effect of BM leptin and adiponectin content on infant FFM and FM deposition through the first 12 months of life: Specifically, they saw an inverse association between the daily intake of adiponectin and the infant fat-free mass but a positive relationship with infant fat mass. At one year, higher intake of BM leptin was associated with higher fat mass deposition. Interestingly, contrasting data were found in this study by Fields et al. [19], where authors observed that milk leptin concentration reduces through lactation and it was higher in obese mothers; furthermore, this hormone shows a negative association with infant length, FM percentage, total FM, and trunk fat at six months of age. In a previous pilot study on 19 exclusively breastfeeding mother–infant dyads [20], Fields observed that greater BM leptin was associated with lower BMI-for-age z-score (BMIZ), and higher content of BM insulin was associated with lower infant weight, BMIZ, and FFM. All these results concerning leptin suggest that this hormone may have a role both with regard to FM and FFM deposition. The relationship between BM adiponectin, leptin, and insulin with infant body composition was assessed in 430 mother–infant dyads taking part into the Canadian Healthy Infant Longitudinal Development (CHILD) Study [21]. Data showed an inverse correlation between BM leptin and insulin content and infant WLZ and BMIZ at four months of age, whereas no significant association was found between BM adiponectin concentrations and infant body composition. Meyer et al. [22] longitudinally investigated the correlation among BM leptin and adiponectin with body composition in children aged three to five years. The authors previously reported a positive association between BM adiponectin concentration and FM and weight gain in infants in the first two years of age. However, the findings of this analysis failed to demonstrate a significant relationship of BM leptin and adiponectin levels, assessed at six weeks and four months after delivery, with later body composition characteristics. Brunner and colleagues [23] investigated the association of leptin and adiponectin in BM with infant weight gain and body composition using skinfold thickness assessment in the first 24 months of life. Although BM leptin assessed at four months was negatively associated with concurrent infant weight, BMI, and FFM, no relationship with infant growth and body composition at later ages was reported. On the contrary, there was a tendency for BM adiponectin to be negatively associated with infant growth parameters in the first four months of age, but afterwards, it was positively associated with infant weight gain and FM up to the first 24 months of life. Mohamad et al. [24], assessed the relationship of maternal serum and BM adipokines with infant adiposity development. A higher BM adiponectin concentration at two months after delivery was associated with reduced infant body weight, BMIZ, and abdominal circumference at two months of age. After adjusting for confounders, BM adiponectin at two months postpartum was the only factor that remained independently associated with infant adiposity at two months of age. This result indicates that BM adiponectin may be protective on infant adiposity development in the early postnatal period. Cesur et al. [25] investigated the relationship between ghrelin and adiponectin content in BM and serum samples of 25 mother–infant pairs with the anthropometry of newborn infants at one and four months of life. BM ghrelin content was significantly higher than the infant and maternal serum ghrelin at both study points. Moreover, the level of the fourth month BM ghrelin level positively correlated with infants’ weight gain throughout the study. Larsson et al. [26] aimed to identify the determinants of early growth by comparing two groups of exclusively breastfed infants, one with excessive weight gain and the second one with a normal growth pattern during the first five months. The lack of difference in the BM intake at the age of five months between the groups suggests that the high weight gain could be driven by factors related to milk composition. Accordingly, the leptin content was significantly decreased in the BM of the group characterized by high weight gain, thus stimulating appetite and milk intake in this group of infants. Anyhow, it has to be considered that, in this study, measurement of BM was carried out only at five months of age and not earlier, when growth velocity was considerably higher. Ucar and colleagues [27] investigated whether leptin may exert an effect on satiety by comparing the formilk and hindmilk leptin concentrations in 18 lactating women. However, no differences were found between the leptin levels of BM samples assessed during the two different phases of feeding at breast. Additionally, no association between leptin concentrations in both BM and maternal plasma and infants’ body weight, BMI, TSF, and left upper arm circumference measurements was found. Concerning the role of adiponectin in BM, a longitudinal observational study of two cohorts of 45 mother–infant pairs was carried out to determine an association between milk adiponectin and infant growth. No significant difference was detected among the two cohorts at the beginning of the study. However, higher BM adiponectin was associated, through the first six months, with lower infant adiposity—in particular, with weight-for-age z-score (WAZ) and WLZ, but not length for age z-score (LAZ), after adjusting for confounders and irrespective of the cohort [28]. Similarly, a negative association between adiponectin and infant growth (i.e., weight-for-height z-score (WHZ) and head circumference) in two cohorts of infants from 48 healthy mothers and from 48 mothers with gestational diabetes mellitus was found [12]. No associations between BM insulin, leptin, and ghrelin concentrations and infant WHZ and head circumference resulted [12]. In Table 1 the main findings of the studies meeting the inclusion criteria are shown.

## 4. Discussion

In this systematic review, we explored updated evidences on five hormones (leptin, ghrelin, IGF-1, adiponectin, insulin) described in BM that are also related with hunger, fat deposition, and adipose tissue metabolism in all stages of life. Leptin is an hormone synthetized by adipose cells and mucosal cells in the small intestine; it regulates energy homeostasis by interacting with its receptors in the hypothalamus, inhibiting hunger [29]. As a result, fat storage in adipocytes is decreased [30]. In newborns, Chaoimh et al. [31] found a negative relationship between cord leptin content and weight gain, demonstrating that a low weight gain is accompanied by a reduced fat mass deposition in the early postnatal period. Accordingly, BM leptin has been reported to be inversely associated with infant global adiposity and trunk fat at six months of age [19]. Moreover, a higher content of BM leptin was associated with lower WLZ and BMIZ in infants [20,21]. However, this effect appears to be temporary, as indicated by the lack of effect of leptin levels in BM on body composition in the first years of age [22,23]. Interestingly, despite this well-known anti-adiposity effect, BM leptin content has also been positively correlated with higher weight gain in infants in the early postnatal period, and increased adiposity as far as 12 months of lactation [16,18]. Consistently with these results, a possible relationship of BM leptin with body fat stores and infant adiposity has been suggested by Savino and colleagues, who determined leptin values in serum and in the mothers’ BM. The authors found positive correlations between BM leptin and infant serum leptin, and between infant serum leptin and both infants’ BMI and weight [32]. Yis et al. also found a positive correlation with leptin serum level in infants, but failed to demonstrate a significant correlation with BM leptin levels [17]. These inconstistent results may pose a doubt on BM leptin’s hormonal role during lactation, which could differ from what is known to be its primary role in human body, as hypothesized by Ucar and colleagues who failed to demonstrate a role played by leptin BM levels as a satiety factor, and also showed no correlation with infants’ weight and adiposity [27]. Opposite to leptin, ghrelin is considered to be the “hunger hormone”. It is a neuropetide secreted by specific gastrointestinal cells when the stomach is empty, that acts on the hypothalamus to stimulate appetite, increase gastric acid secretion, and improve gastrointestinal motility [33]. A different relationship between the serum content of hormones involved in satiety and appetite regulation, including ghrelin, has been found by Vásquez-Garibay et al. [34]. Specifically, the authors demonstrated a more significant association in breastfeeding mother–infant pairs than in formula-fed ones. Moreover, Cesur et al. found that ghrelin in BM is significantly higher than serum ghrelin, both in the infants’ and the mothers’ samples through first months of lactation [25]. However, although there is agreement regarding the role of breastfeeding in modulating the mechanisms underlying satiety and appetite regulation, studies available in the literature on BM ghrelin and infants’ weight gain have shown inconsistent results. While Kon and colleagues [16] reported a positive correlation between BM ghrelin in the first two months of lactation with higher weight gain in infants, the results were not confirmed by Yis et al., despite again finding a positive correlation with infants’ serum ghrelin levels and TSF [17]. Equally to ghrelin, adiponectin is a peptide with hormonal functions that stimulate hunger, acting on the hypothalamus. It is produced specifically by the adipose tissue, and its serum levels are inversely correlated with the FM percentage of the body in the adult population [35]. Its levels in BM and association with infants’ FM and growth have been explored by many studies included in this review, with inconsistent results. Young et al. inversely correlated BM adiponectin levels with FM gain in healthy term infants, consistent with its hormonal function [12], whereas Gridneva and colleagues found a correlation with a higher adiposity in the first year of life [18]. Similar results, both with a positive or negative correlation, were obtained in all other studies explored, whereas the largest cohort CHILD study failed to find any association between adiponectin BM levels and infants’ body composition [21]. IGF-1 is part of countless metabolic pathways of cells’ signaling with their environment, often referred as IGF “axis” that plays a primary role in cell proliferation and inhibition of cell death (apoptosis) in both physiological and pathological states [36]. IGF-1 expression is also required for achieving maximal growth in developing organisms [37]. In this review, we found only one study that explored IGF-1 BM levels and infants’ growth. Kon et al. reported a higher BM level of IGF-1 in those mothers whose infants showed a higher weight gain [16]. Insulin is a peptide that acts as a hormone, produced by specific endocrine cells located in the pancreatic tissue. It is considered to be the most important anabolic hormone and promotes cellular intake glucose in muscle and adipose tissue [38]. According to these functions, its levels in BM have been demonstrated to positively correlate with infant WLZ trajectory in the first months of life [12]. However, similarly to other hormones listed above, these findings were not confirmed by other studies considered in this review. However, limitations should be noted when interpreting the results. First of all, more longitudinal investigations are needed, especially during the early lactation period, to clarify the effects of breast milk hormones on growth and regulation of nutrition in infancy and childhood. The use of different outcomes to measure the child’s growth was a further limitation of the review. The duration of the studies was until different ages and in some studies, it was not possible to arrange clinical visits before the age of five to six months. Finally, infant growth was evaluated using anthropometric measurements: More accurate measurements of body composition will help accurately evaluate the mediation effect of hormones in BM.

## 5. Conclusions

The inconsistent findings of the studies considered in this review could be explained with the concomitant action of countless factors interfering with infants’ body composition during the first years of life. While some of these, including basic subject characteristics, could be identified and controlled, other factors such as synergetic or antagonist functions of many other bioactive components of BM, that could play a role in modeling growth and FM apposition, are difficult—if not impossible—to take into account. Although further studies are needed to determine the specific role of hormones and adipokines in BM, undoubtedly, these bioactive components participate in metabolic pathways related to body composition. The understanding of these pathways could help gain further insight on infants’ growth, both in physiological and pathological settings.

## Figures and Tables

**Table 1:** Observational studies on hormones’ dosage in breast milk and infants’ growth and anthropometry.

Study	Sample Size	Growth and Anthropometric Outcomes	Hormones in Breast Milk	Major Findings
[12]	96 BF infants	Infant body weight (kg), length (cm), WLZ, head circumference (cm).	Adiponectin, leptin, insulin and ghrelin	Adiponectin inversely associated with WLZ and head circumference (*p* ≤ 0.003). Association between adiponectin and insulin and head circumference (*p* ≤ 0.007 and *p* ≤ 0.049, respectively).
[15]	41 BF infants	WLZ, % body fat from skinfolds from 0 to 4 mo.	Leptin, adiponectin, ghrelin, insulin	Negative association between HM insulin and WLZ trajectory in infants of normal weight mothers (*p* = 0.028)
[16]	103 BF infants	Weight gain (g/mo).	Leptin, adiponectin, ghrelin, IGF-1	Correlation between breast milk IGF-1 and infant weight gain (r = 0.294, *p* = 0.043). Higher ghrelin levels at 1 and 2 mo and higher leptin levels at 2 and 3 mo of lactation (*p* < 0.05) in infants with high weight gain (>1000 g/mo).
[17]	24 BF infants	Infant’s body weight (g), length (cm), triceps skinfold thickness (mm), postnatal weight gain (g) at 3 and 6 mo of age.	Ghrelin, leptin	No correlation between breast-milk ghrelin or breast-milk leptin with anthropometric data.
[18]	20 BF infants	Infant’s body weight (g), length (cm), BMI (kg/m^2^), ultrasound skinfolds, bioimpedance spectroscopy and FMI (FM (kg)/length (m)^2^), FFMI (FFM (kg)/length (m)^2^).	Adiponectin, leptin	Higher intake of adiponectin associated with lower infant FFM (*p* = 0.005) and FFM index (*p* = 0.009) and higher FM (*p* < 0.001), FM index (FMI; *p* < 0.001), and %FM (*p* < 0.001). Higher intake of leptin associated with larger increases in infant adiposity (2–12 month): FM, *p* = 0.0006; %FM, *p* = 0.0004.
[19]	37 BF infants	Infant’s body weight (g), length (cm), FM (g and %), FFM (g), trunk fat mass (g).	Insulin, leptin	Inverse association between leptin levels at 1 mo and infant length (*p* = 0.0257), FM % (*p* = 0.0223), FM (g) (*p* = 0.0226), and trunk fat mass (*p* = 0.0111) at 6 mo.
[20]	19 BF infants	Infant’s body weight (g), length (cm), WLZ, BMIZ, FM (g and %), FFM (g), trunk fat mass (g) at 1 mo of age	Leptin, insulin	Leptin associated with lower BMIZ (r = −0.54, *p* = 0.03). Higher concentrations of insulin associated with lower infant weight, relative weight, and FFM (r = −0.49–0.58, *p* < 0.06).
[21]	430 BF infants	Infant’s body weight (g), length (cm), WFL, BMIZ at 4 mo and 1 yrs of age.	Adiponectin, leptin, insulin	Higher leptin associated with lower infant WLZ at 4 mo (β − 0.67, 95% confidence interval (CI): −1.17, −0.17 for highest vs lowest quintile) and 1 yrs (β − 0.58, 95% CI: −1.02, −0.14). Insulin showed a U-shaped association, with intermediate concentrations predicting the lowest infant WLZ at 4 mo (β − 0.51, 95% CI: −0.87, −0.15 for third vs lowest quintile) and 1 yrs (β − 0.35, 95% CI: −0.66, −0.04). Adiponectin not associated with infant body composition.
[22]	147 BF infants	Infant’s body weight (kg), BMI percentiles, sum of four skinfolds (mm), FM (kg and %), FFM (kg) at 3, 4, and 5 yrs of age.	Adiponectin, leptin	No association between leptin or total adiponectin levels assessed at 6 weeks post-delivery with children’s body weight, BMI percentiles, sum of four skinfolds, measurements, FM (kg and %), or FFM (kg).
[23]	188 BF infants	The relationship of BM leptin and adiponectin with infant weight gain and body composition up to the age of 2 yrs.	Adiponectin, leptin	Milk leptin at 4 mo negatively associated with infant weight ([95%CI]: −604.96 g [−1166.19; −43.72], *p* = 0.037) and FFM (−400.95 g [−777.64; −24.25], *p* = 0.039) at the age of 4 mo. Adiponectin tended to be negatively associated with infant FFM (*p* = 0.015) and weight (*p* = 0.054) in the first 4 mo, but afterwards was positively related to weight gain (*p* = 0.027) and the sum of skinfolds (*p* = 0.047) up to 2 years.
[24]	155 BF infants	Infant’s body weight (kg) and BMIZ, abdominal circumference (cm).	Leptin, adiponectin	The higher level of adiponectin at 2 mo postpartum associated with reduced infant body weight (β = −0.54 *p* = 0.003), BMIZ (β = −0.79, *p* = 0.008) and abdominal circumference at 2 mo of age (β = −2.34, *p* = 0.003). No association between adiponectin at birth and 2 mo with infant adiposity at 6 and 12 mo of age. An increased maternal ALR was related to reduced infant BMIZ at birth.
[25]	25 BF infants	Infant’s body weight (g), length (cm), BMI (kg/m^2^).	Ghrelin, adiponectin	Positive correlation between the level of the 4th mo ghrelin level and infants’ weight gain (r = 0.51, *p* = 0.025).
[26]	30 BF infants	Infant’s body weight (g), length (cm), WAZ, LAZ, BAZ, TSFZ, SSFZ.	Leptin, adiponectin	A 40% reduction of median leptin content at 5 mo in the high weight gain group (*p* = 0.045). At 5 mo, no significant associations between milk concentrations of hormones and infants’ WAZ, BAZ or LAZ, or energy and hormones and infant’s anthropometry (WAZ, BAZ, or LAZ) or change in these z-scores from birth to the 5 mo visit (all *p* > 0.11).
[27]	18 BF infants	Infant’s body weight (kg), BMI (kg/m^2^), triceps skinfold thickness (mm), left upper arm circumference (mm).	Leptin	No correlation between Log leptin concentrations and infants’ body weight, BMI, triceps skinfold thickness, and left upper arm circumference measurements (*p* > 0.05).
[22]	322 BF infants	Infant’s body weight (kg), length (cm), WAZ, BMI (kg/m^2^), LAZ, WLZ.	Adiponectin	During the first 6 months, higher adiponectin associated with lower infant WAZ (β = 0.20 ± standard error (SE) 0.04, *p* = 0.0001) and WLZ (β = 0.29 ± 0.08, *p* = 0.0002) Adiponectin not associated with infant length.

Mo = months; yrs = years; BF = breastfed; FM = fat mass; FFM = fat-free mass; FMI = FM index; FFMI = FFM index; BMI = body mass index; WAZ = weight for age z-score; WLZ = weight-for-length/height z-score; LAZ = length-for-age *z*-score; BMIZ = BMI z-score; TSFZ = triceps skinfold-for-age z-score; SSFZ = subscapular skinfold-for-age; ALR = maternal adiponectin to leptin ratio; β = beta regression coefficient.
